# Supplementary material for: Using Background Knowledge from Preceding Studies for Building a Random Forest Prediction Model: A Plasmode Simulation Study
Source: Entropy (Basel). 2022 Jun 20;24(6):847. doi: 10.3390/e24060847 (PMC9222226; doi:10.3390/e24060847)
Supplement: Supplementary file 1 [file entropy-24-00847-s001.zip › entropy-1727795-supplementary.pdf]

Supplemental material:

# Using background knowledge from preceding studies for building a random forest prediction model: A plasmode simulation study

**Lorena Hafermann [1], Nadja Klein[2,*], Geraldine Rauch[1], Michael Kammer [3] and Georg Heinze [3,*]**

[1] Institute of Biometry and Clinical Epidemiology, Charité–Universitätsmedizin Berlin, Corporate Member of Freie Universität Berlin and Humboldt-Universität zu Berlin, Charitéplatz 1, 10117 Berlin,
Germany; lorena.hafermann@charite.de (L.H.); geraldine.rauch@tu-berlin.de (G.R.)

[2] Chair of Statistics and Data Science, School of Business and Economics, Humboldt-Universität zu Berlin, Unter den Linden 6, 10099 Berlin, Germany

[3] Section for Clinical Biometrics, Center for Medical Statistics, Informatics and Intelligent Systems, Medical University of Vienna, Spitalgasse 23, 1090 Vienna, Austria; michael.kammer@meduniwien.ac.at

* Correspondence: nadja.klein@hu-berlin.de (N.K.), georg.heinze@meduniwien.ac.at (G.H.)

# Section S1: Treatment discontinuation study – Study description, meta data and data dictionary

## Background of study

PUKO-BHD was a nationwide pharmacoepidemiologic study, using data from Austria, conducted at the Medical University of Vienna in which the costs, overutilization and comparative effectiveness of branded and generic medications for the treatment of the indications arterial hypertension, hyperlipidemia and hyperglycemia were investigated. Three publications and a dissertation thesis [1-4] describe the results of this study.

In this subproject, the continuity of treatment after a first prescription of lisinopril (ATC code C09AA03; see also https://www.whocc.no/atc_ddd_index/) was investigated. In particular, the filling of a follow-up prescription within 6 weeks from the first prescription of a package of lisinopril was considered as treatment continuation, while no such follow-up filling constituted treatment discontinuation. In particular, the objective of this subproject is to fit a prediction model to prognosticate treatment discontinuation at the time of first prescription. Such a model would be useful to identify patients at high risk of treatment discontinuation, and may contribute to save unnecessary costs of health care.

## Inclusion criteria

Individuals who had an active social insurance contract at least once between 2007 and 2012 with the nine provincial sickness funds and the four nationwide sickness funds for state employees, farmers, self-employed and railway workers were eligible for this study. (All sickness funds contributed all their data from 2009 to 2012, but only some of the sickness funds also contributed data for the years 2007 and 2008.) Since social insurance is mandatory in Austria, these thirteen sickness funds cover most of the population (about 97%). Persons were included in this study if they received a prescription for lisinopril in the study period, had been under observation for at least 180 days before the index prescription and 44 days after the index prescription, and had not filled a prescription for lisinopril during the 180 days preceding the index prescription. Patients who died within 44 days from the index prescription were excluded.

Since the data stem from Subproject 3 (comparative effectiveness), prescriptions were only included if at the time of prescription both generic and branded versions were available at the same combination of strength (dose of medication per pill) and volume (number of pills per package).

## Outcome variable

The binary outcome variable was treatment discontinuation, defined as 'event' and coded as 1 if a patient filled a follow-up prescription for lisinopril within 44 days from the index prescription, and as 'non-event' (code 0) otherwise.

## Potential predictors

As potential predictors, demographic data at the index prescription, descriptors of the index prescription, and covariates evaluated in two covariate harvesting windows ('ante1' period starting 14 days before the index prescription, and 'ante2' period starting 180 days before the index prescription and ending 15 days before the index prescription) were considered.

## Data dictionary

| Group | Variable | Variable names | Codes |
|---|---|---|---|
| Demographic | Age at index prescription in years | age | Numeric, integer |
| | Squared age: (age/100)^2 | age2 | Numeric |
| | Sex | female | 1=female, 0=male |
| | Copayment waiver status, expressed as the proportion of filled prescription with copayment waived in the year of the index prescription. (In Austria, copayment waiver status can be permanent or dynamic depending on number of prescriptions filled relative to income.) | waive_rate | Numeric [0,1] |
| Descriptors of index prescription | Type of medication (branded or generic) | branded | 1=branded, 0=generic |
| | Year of index prescription | year | Integer (2007 to 2012) |
| | Sickness funds ID | 13 dummy variables (one hot coding): vtr_id_dum.vtr_id_facXX where XX=5, 7, 11, 12, 13, 14, 15, 16, 17, 18, 19, 40, 50 | 0, 1 |
| | Specialty of prescriber: general practitioner, internal medicine specialist, hospital, other | 4 dummy variables (one hot coding): disc_id_dum.disc_id1, disc_id_dum.disc_id_fac2, disc_id_dum.disc_id_fac3, disc_id_dum.disc_id_fac4 | 0, 1 |
| | Strength–volume combination. Six combinations with frequency of both generic and branded prescriptions greater than 100 in the study period were considered. These were the six combinations of strength—5mg, 10mg, and 20mg—and volume—28 and 56 pills. | 6 dummy variables (one hot coding): f.package2, f.package3, f.package6, f.package7, f.package9, f.package10 | 0, 1 |
| Covariates from harvesting window 'ante1' (day -14 to day 0 from index prescription | Hospital admission (no, yes) | ante1_kha | 0, 1 |

| | | | |
|---|---|---|---|
| | Number of days in hospital > 14 (no, yes) | ante1_kha14 | 0, 1 |
| | Hospital discharge diagnoses as ICD10 codes | ante1_is_XXX where XXX is an ICD10 code (e.g., XXX=a04 is bacterial intestinal infection). Subgroups were summarized into 3-digit codes. ICD10 codes can be found, e.g., at https://icd.who.int/browse10/2019/en | 0, 1 |
| | Filled prescriptions as ATC level 2 code | ante1_is_XXX_mg,<br>ante1_is_XXX_ml,<br>ante1_is_XXX_mg,<br>ante1_is_XXX_mg_ml,<br>ante1_is_XXX_iu, or<br>ante1_is_XXX_pct<br>(depending on dosage form), where XXX is a 3-digit ATC-level-2 code. See https://www.whocc.no/atc_ddd_index/ for ATC codes.<br>E.g., ante1_is_a02_mg describes prescription of a drug for acid-related disorders in dosage form 'mg', e.g., the proton pump inhibitor omeprazole 20mg. | 0, 1 |
| Covariates from harvesting window 'ante2' (day -180 to day -15 from index prescription) | Hospital admission (no, yes) | ante2_kha | 0, 1 |
| | Number of days in hospital > 14 (no, yes) | ante2_kha14 | 0, 1 |
| | Hospital discharge diagnoses as ICD10 codes | ante2_is_XXX | 0, 1 |
| | Filled prescriptions as ATC level 2 code | ante2_is_XXX_mg, etc. | 0, 1 |

## References

[1] Heinze, G., Hronsky, M., Reichardt, B., Baumgärtel, C., Müllner, M., Bucsics, A. and Winkelmayer, W. C. (2014). Potential Savings in Prescription Drug Costs for Hypertension, Hyperlipidemia, and Diabetes Mellitus by Equivalent Drug Substitution in Austria: A Nationwide Cohort Study. Applied Health Economics and Health Policy 13, 193–205.

[2] Heinze, G., Jandeck, L. M., Hronsky, M., Reichardt, B., Baumgärtel, C., Bucsics, A., Müllner, M. and Winkelmayer, W. C. (2015). Prevalence and determinants of unintended double medication of antihypertensive, lipid-lowering, and hypoglycemic drugs in Austria: a nationwide cohort study. Pharmacoepidemiology and Drug Safety 25, 90–99.

[3] Tian, Y., Reichardt, B., Dunkler, D., Hronsky, M., Winkelmayer, W. C., Bucsics, A., Strohmaier, S. and Heinze, G. (2020). Comparative effectiveness of branded vs. generic versions of antihypertensive, lipid-lowering and hypoglycemic substances: a population-wide cohort study. Scientific Reports 10. doi:10.1038/s41598-020-62318-y.

[4] Jandeck, L.M. Populationsweite Utilisationsuntersuchung in den chronischen Krankheitsbildern Hypertonie, Hyperlipidämie und Typ 2 Diabetes Mellitus. Inaugural-Dissertation, Ruhr-Universität Bochum, 2014.

Table S1: Motivating study: permutation-based predictor importance for the 20 most important predictors scaled by 1000 for improved readability. Importance is computed as the increase in mean prediction error in out-of-bag observations by permuting a predictor.

| **Predictor** | **Importance (x 1000)** |
|---|---|
| waive_rate | 6.56 |
| f.package2 | 4.43 |
| ante1_kha | 3.52 |
| ante1_is_i10 | 2.93 |
| f.package9 | 2.62 |
| f.package3 | 2.24 |
| age | 1.80 |
| ante2_is_i10 | 1.80 |
| f.package6 | 1.70 |
| ante2_kha14 | 1.67 |
| ante1_kha14 | 1.49 |
| f.package7 | 1.46 |
| ante2_is_c07_mg | 1.19 |
| ante2_is_b01_mg | 0.99 |
| ante2_is_c10_mg | 0.89 |
| ante2_is_c01_mg | 0.87 |
| ante2_is_n05_mg | 0.84 |
| ante2_is_c08_mg | 0.81 |
| ante2_is_c03_mg | 0.79 |
| ante1_is_i25 | 0.75 |

Table S2: Simulation study: mean calibration slope. M1: no preselection; M2: preselection based on Lasso; M3: preselection based on intersection of Lasso and univariate selection; M4: preselection based on union of Lasso and univariate selection; M5: preselection based on optimum of the Lasso and univariate model. Results are based on 1000 replications. Bold numbers indictate optimal model in a scenario.

| **Predictability** | **Sample Size** | **Lasso** | **M1** | **M2** | **M3** | **M4** | **M5** |
|---|---|---|---|---|---|---|---|
| **Strong** | 4000 | 1.13 | 1.29 | 1.22 | **1.11** | 1.17 | 1.14 |
| | 2000 | 1.15 | 1.32 | 1.21 | **1.07** | 1.14 | 1.09 |
| | 1000 | 1.14 | 1.37 | 1.19 | 1.03 | 1.11 | **1.02** |
| | 500 | 1.14 | 1.44 | 1.16 | **0.99** | 1.06 | 0.92 |
| | 250 | 1.16 | 1.52 | 1.11 | **0.99** | **0.99** | 0.91 |
| **Weak** | 4000 | 1.22 | **1.03** | 0.95 | 0.85 | 0.86 | 0.81 |
| | 2000 | 1.21 | **1.00** | 0.85 | 0.79 | 0.76 | 0.70 |
| | 1000 | 1.22 | **0.94** | 0.75 | 0.79 | 0.65 | 0.58 |
| | 500 | 1.20 | **0.87** | 0.66 | 0.84 | 0.53 | 0.49 |
| | 250 | **1.02** | 0.80 | 0.55 | 1.04 | 0.43 | 0.41 |

Table S3: Simulation study: standard deviation of calibration slope. M1: no preselection; M2: preselection based on Lasso; M3: preselection based on intersection of Lasso and univariate selection; M4: preselection based on union of Lasso and univariate selection; M5: preselection based on optimum of the Lasso and univariate model. Results are based on 1000 replications.

| **Predictability** | **Sample Size** | **Lasso** | **M1** | **M2** | **M3** | **M4** | **M5** |
|---|---|---|---|---|---|---|---|
| Strong | 4000 | 0.0647 | 0.0476 | 0.0493 | 0.0413 | 0.0464 | 0.0458 |
| | 2000 | 0.0991 | 0.0633 | 0.0642 | 0.0544 | 0.0599 | 0.0538 |
| | 1000 | 0.1460 | 0.0819 | 0.0852 | 0.0697 | 0.0783 | 0.0671 |
| | 500 | 0.2034 | 0.1156 | 0.1122 | 0.1034 | 0.1031 | 0.0868 |
| | 250 | 0.3202 | 0.1538 | 0.1530 | 0.1766 | 0.1287 | 0.1430 |
| Weak | 4000 | 0.1547 | 0.0665 | 0.0706 | 0.0715 | 0.0609 | 0.0556 |
| | 2000 | 0.2496 | 0.0752 | 0.0783 | 0.0972 | 0.0666 | 0.0583 |
| | 1000 | 0.4765 | 0.0873 | 0.1028 | 0.2053 | 0.0801 | 0.0807 |
| | 500 | 0.7642 | 0.0983 | 0.2281 | 0.6868 | 0.1046 | 0.1269 |
| | 250 | 0.9989 | 0.1240 | 1.5353 | 12.9081 | 0.2284 | 0.1911 |

Table S4: Simulation study: mean MSE of log calibration slope. M1: no preselection; M2: preselection based on Lasso; M3: preselection based on intersection of Lasso and univariate selection; M4: preselection based on union of Lasso and univariate selection; M5: preselection based on optimum of the Lasso and univariate model. Results are based on 1000 replications. Bold numbers indictate optimal model in a scenario.

| Predictability | Sample Size | Lasso | M1 | M2 | M3 | M4 | M5 |
|---|---|---|---|---|---|---|---|
| **Strong** | 4000 | 0.019 | 0.064 | 0.040 | **0.011** | 0.026 | 0.018 |
| | 2000 | 0.026 | 0.080 | 0.039 | **0.007** | 0.020 | 0.010 |
| | 1000 | 0.032 | 0.101 | 0.035 | **0.005** | 0.015 | **0.005** |
| | 500 | 0.045 | 0.136 | 0.030 | **0.011** | 0.012 | 0.017 |
| | 250 | 0.087 | 0.182 | 0.027 | 0.031 | **0.017** | 0.034 |
| **Weak** | 4000 | 0.052 | **0.005** | 0.009 | 0.037 | 0.029 | 0.051 |
| | 2000 | 0.069 | **0.006** | 0.036 | 0.072 | 0.082 | 0.137 |
| | 1000 | 0.120 | **0.013** | 0.109 | 0.135 | 0.206 | 0.318 |
| | 500 | 0.222 | **0.033** | 0.405 | 1.161 | 0.463 | 0.769 |
| | 250 | 0.741 | **0.080** | 2.456 | 5.897 | 1.818 | 2.344 |

Table S5: Simulation study: standard deviation of MSE of log calibration slope. M1: no preselection; M2: preselection based on Lasso; M3: preselection based on intersection of Lasso and univariate selection; M4: preselection based on union of Lasso and univariate selection; M5: preselection based on optimum of the Lasso and univariate model. Results are based on 1000 replications.

| Predictability | Sample Size | Lasso | M1 | M2 | M3 | M4 | M5 |
|---|---|---|---|---|---|---|---|
| **Strong** | 4000 | 0.0147 | 0.0187 | 0.0161 | 0.0076 | 0.0127 | 0.0106 |
| | 2000 | 0.0251 | 0.0268 | 0.0200 | 0.0079 | 0.0145 | 0.0091 |
| | 1000 | 0.0384 | 0.0376 | 0.0254 | 0.0075 | 0.0152 | 0.0065 |
| | 500 | 0.0615 | 0.0585 | 0.0303 | 0.0169 | 0.0171 | 0.0209 |
| | 250 | 0.1235 | 0.0856 | 0.0389 | 0.0554 | 0.0253 | 0.0417 |
| **Weak** | 4000 | 0.0532 | 0.0067 | 0.0133 | 0.0318 | 0.0229 | 0.0306 |
| | 2000 | 0.0984 | 0.0083 | 0.0352 | 0.0643 | 0.0492 | 0.0622 |
| | 1000 | 0.2460 | 0.0176 | 0.0916 | 0.3069 | 0.1089 | 0.1476 |
| | 500 | 0.4278 | 0.0368 | 4.1965 | 10.2555 | 0.2962 | 4.1922 |
| | 250 | 6.6466 | 0.1307 | 15.0202 | 24.6343 | 10.2170 | 11.8415 |

Table S6: Simulation study: mean of maximum contribution to cross-entropy. M1: no preselection; M2: preselection based on Lasso; M3: preselection based on intersection of Lasso and univariate selection; M4: preselection based on union of Lasso and univariate selection; M5: preselection based on optimum of the Lasso and univariate model. Results are based on 1000 replications. Bold numbers indictate optimal model in a scenario.

| **Predictability** | **Sample Size** | **Lasso** | **M1** | **M2** | **M3** | **M4** | **M5** |
|---|---|---|---|---|---|---|---|
| **Strong** | 4000 | 4.45 | **2.75** | 3.15 | 3.42 | 3.22 | 3.16 |
| | 2000 | 4.39 | **2.67** | 3.02 | 3.35 | 3.25 | 3.32 |
| | 1000 | 4.35 | **2.57** | 2.93 | 3.30 | 3.24 | 3.47 |
| | 500 | 4.19 | **2.48** | 2.82 | 3.05 | 3.20 | 3.55 |
| | 250 | 3.89 | **2.39** | 2.70 | 2.73 | 3.17 | 3.07 |
| **Weak** | 4000 | 1.92 | **1.88** | 2.10 | 2.39 | 2.35 | 2.49 |
| | 2000 | 1.94 | **1.81** | 2.10 | 2.28 | 2.35 | 2.52 |
| | 1000 | 1.91 | **1.75** | 2.10 | 2.06 | 2.37 | 2.46 |
| | 500 | 1.85 | **1.71** | 2.01 | 1.71 | 2.31 | 2.34 |
| | 250 | 1.88 | **1.67** | 1.91 | 1.76 | 2.16 | 2.14 |

Table S7: Simulation study: standard deviation of maximum contribution to cross-entropy. M1: no preselection; M2: preselection based on Lasso; M3: preselection based on intersection of Lasso and univariate selection; M4: preselection based on union of Lasso and univariate selection; M5: preselection based on optimum of the Lasso and univariate model. Results are based on 1000 replications.

| **Predictability** | **Sample Size** | **Lasso** | **M1** | **M2** | **M3** | **M4** | **M5** |
|---|---|---|---|---|---|---|---|
| **Strong** | 4000 | 0.6443 | 0.1460 | 0.2626 | 0.3142 | 0.2652 | 0.2500 |
| | 2000 | 0.8005 | 0.1460 | 0.2695 | 0.3187 | 0.2840 | 0.2999 |
| | 1000 | 0.9213 | 0.1604 | 0.2803 | 0.3653 | 0.3373 | 0.3796 |
| | 500 | 1.1765 | 0.1955 | 0.3171 | 0.4516 | 0.4047 | 0.4640 |
| | 250 | 1.4494 | 0.2295 | 0.3852 | 0.6309 | 0.4790 | 0.4385 |
| **Weak** | 4000 | 0.3548 | 0.1364 | 0.2239 | 0.3008 | 0.2539 | 0.2756 |
| | 2000 | 0.4332 | 0.1434 | 0.2479 | 0.3278 | 0.3017 | 0.3378 |
| | 1000 | 0.5800 | 0.1408 | 0.3034 | 0.4283 | 0.3375 | 0.3694 |
| | 500 | 0.7526 | 0.1433 | 0.4304 | 0.6855 | 0.3602 | 0.4224 |
| | 250 | 0.9051 | 0.1583 | 0.6158 | 0.8520 | 0.4438 | 0.4899 |

Table S8: Simulation study: standard deviation of cross-entropy. M1: no preselection; M2: preselection based on Lasso; M3: preselection based on intersection of Lasso and univariate selection; M4: preselection based on union of Lasso and univariate selection; M5: preselection based on optimum of the Lasso and univariate model. Results are based on 1000 replications.

| Predictability | Sample Size | Lasso | M1 | M2 | M3 | M4 | M5 |
|---|---|---|---|---|---|---|---|
| **Strong** | 4000 | 49 | 45 | 20 | 27 | 48 | 50 |
| | 2000 | 54 | 47 | 28 | 34 | 54 | 53 |
| | 1000 | 68 | 53 | 42 | 52 | 59 | 64 |
| | 500 | 91 | 65 | 68 | 88 | 75 | 79 |
| | 250 | 157 | 91 | 118 | 166 | 115 | 130 |
| **Weak** | 4000 | 25 | 25 | 22 | 26 | 30 | 33 |
| | 2000 | 29 | 28 | 29 | 36 | 35 | 38 |
| | 1000 | 42 | 32 | 45 | 59 | 51 | 57 |
| | 500 | 57 | 42 | 85 | 121 | 85 | 101 |
| | 250 | 89 | 64 | 165 | 252 | 136 | 195 |

Table S9: Simulation study: standard deviation of AUROC. M1: no preselection; M2: preselection based on Lasso; M3: preselection based on intersection of Lasso and univariate selection; M4: preselection based on union of Lasso and univariate selection; M5: preselection based on optimum of the Lasso and univariate model. Results are based on 1000 replications.

| Predictability | Sample Size | Lasso | M1 | M2 | M3 | M4 | M5 |
|---|---|---|---|---|---|---|---|
| **Strong** | 4000 | 0.0047 | 0.0045 | 0.0021 | 0.0028 | 0.0046 | 0.0048 |
| | 2000 | 0.0052 | 0.0048 | 0.0031 | 0.0035 | 0.0052 | 0.0050 |
| | 1000 | 0.0065 | 0.0052 | 0.0045 | 0.0053 | 0.0057 | 0.0060 |
| | 500 | 0.0090 | 0.0060 | 0.0074 | 0.0097 | 0.0076 | 0.0072 |
| | 250 | 0.0149 | 0.0077 | 0.0158 | 0.0238 | 0.0116 | 0.0134 |
| **Weak** | 4000 | 0.0064 | 0.0055 | 0.0050 | 0.0053 | 0.0060 | 0.0066 |
| | 2000 | 0.0072 | 0.0061 | 0.0069 | 0.0075 | 0.0069 | 0.0074 |
| | 1000 | 0.0115 | 0.0075 | 0.0109 | 0.0139 | 0.0093 | 0.0102 |
| | 500 | 0.0175 | 0.0102 | 0.0199 | 0.0235 | 0.0139 | 0.0157 |
| | 250 | 0.0210 | 0.0156 | 0.0273 | 0.0266 | 0.0220 | 0.0213 |

Table S10: Simulation study: standard deviation of Brier score. M1: no preselection; M2: preselection based on Lasso; M3: preselection based on intersection of Lasso and univariate selection; M4: preselection based on union of Lasso and univariate selection; M5: preselection based on optimum of the Lasso and univariate model. Results are based on 1000 replications.

| Predictability | Sample Size | Lasso | M1 | M2 | M3 | M4 | M5 |
|---|---|---|---|---|---|---|---|
| **Strong** | 4000 | 0.0020 | 0.0017 | 0.0008 | 0.0010 | 0.0020 | 0.0020 |
| | 2000 | 0.0022 | 0.0021 | 0.0011 | 0.0013 | 0.0022 | 0.0121 |
| | 1000 | 0.0028 | 0.0019 | 0.0017 | 0.0020 | 0.0023 | 0.0165 |
| | 500 | 0.0036 | 0.0023 | 0.0027 | 0.0034 | 0.0029 | 0.0237 |
| | 250 | 0.0060 | 0.0044 | 0.0049 | 0.0067 | 0.0045 | 0.0342 |
| **Weak** | 4000 | 0.0012 | 0.0012 | 0.0010 | 0.0012 | 0.0014 | 0.0015 |
| | 2000 | 0.0014 | 0.0013 | 0.0014 | 0.0016 | 0.0016 | 0.0017 |
| | 1000 | 0.0019 | 0.0015 | 0.0020 | 0.0026 | 0.0023 | 0.0025 |
| | 500 | 0.0025 | 0.0020 | 0.0032 | 0.0043 | 0.0036 | 0.0043 |
| | 250 | 0.0036 | 0.0030 | 0.0056 | 0.0078 | 0.0056 | 0.0070 |
